# Seasonal Diet and Prey Preference of the African Lion in a Waterhole-Driven Semi-Arid Savanna

**DOI:** 10.1371/journal.pone.0055182

**Published:** 2013-02-06

**Authors:** Zeke Davidson, Marion Valeix, Freya Van Kesteren, Andrew J. Loveridge, Jane E. Hunt, Felix Murindagomo, David W. Macdonald

**Affiliations:** 1 Wildlife Conservation Research Unit, Recanati-Kaplan Centre, Department of Zoology, Oxford University, Tubney House, Abingdon, United Kingdom; 2 Laboratoire de Biométrie et Biologie Evolutive, CNRS UMR 5558, Université Claude Bernard - Lyon 1, Bât Gregor Mendel, Villeurbanne, France; 3 Zimbabwe Parks and Wildlife Management Authority, Causeway, Harare, Zimbabwe; Australian Wildlife Conservancy, Australia

## Abstract

Large carnivores inhabiting ecosystems with heterogeneously distributed environmental resources with strong seasonal variations frequently employ opportunistic foraging strategies, often typified by seasonal switches in diet. In semi-arid ecosystems, herbivore distribution is generally more homogeneous in the wet season, when surface water is abundant, than in the dry season when only permanent sources remain. Here, we investigate the seasonal contribution of the different herbivore species, prey preference and distribution of kills (i.e. feeding locations) of African lions in Hwange National Park, Zimbabwe, a semi-arid African savanna structured by artificial waterholes. We used data from 245 kills and 74 faecal samples. Buffalo consistently emerged as the most frequently utilised prey in all seasons by both male (56%) and female (33%) lions, contributing the most to lion dietary biomass. Jacobs’ index also revealed that buffalo was the most intensively selected species throughout the year. For female lions, kudu and to a lesser extent the group “medium Bovidae” are the most important secondary prey. This study revealed seasonal patterns in secondary prey consumption by female lions partly based on prey ecology with browsers, such as giraffe and kudu, mainly consumed in the early dry season, and grazers, such as zebra and suids, contributing more to female diet in the late dry season. Further, it revealed the opportunistic hunting behaviour of lions for prey as diverse as elephants and mice, with elephants taken mostly as juveniles at the end of the dry season during droughts. Jacobs’ index finally revealed a very strong preference for kills within 2 km from a waterhole for all prey species, except small antelopes, in all seasons. This suggested that surface-water resources form passive traps and contribute to the structuring of lion foraging behaviour.

## Introduction

Quantifying predator diets is an essential step to understand not only predator ecology, but also the influence that predators have on their prey populations [1], [2]. Recent advances in GPS technology has allowed non invasive carcass observations and faecal analysis to gain increasing knowledge on large mammalian carnivores diet [3], [4], and has also permitted to study the spatial distribution of kills providing important information for predator-prey relationships [5]. Across-ecosystems, comparisons of large mammalian carnivore diet have now provided a good understanding of the preferred prey weight range of several carnivore species [6]–[8], but local studies are still needed to unravel the role of environmental factors and prey availability.

Semi-arid ecosystems are characterized by seasonal variations in surface-water and vegetation resources, with several implications for herbivores, and ultimately their predators. In African semi-arid savannas, surface-water is a heterogeneously distributed, limiting resource which becomes scarcer as the dry season progresses. As such, the regular need to access drinking water constrains the movement of herbivores, and hence their distribution in the dry season [9]–[12]. Water dependence varies between herbivore species [11], [13] but most species need to access drinking water on a few-day basis at the peak of the dry season, and herbivore aggregations frequently form around permanent water sources during dry seasons, as non-permanent sources further afield dry up [14], [15]. Contrastingly during wet seasons, pools of water are more available throughout the landscape and herbivore distributions more homogeneous and dispersed. Seasonal surface-water dynamics thus, influences the probability which predators can find prey and is likely to have several consequences for the ecology of predator-prey interactions.

Additionally, dry season scarcity of good quality forage, exacerbated by local depletion around water sources, tends to make certain herbivores more vulnerable to predation, particularly during low rainfall periods [16]–[19]. Aggregation of vulnerable herbivores around water sources in the dry season is likely to attract predators. The vulnerability to predation is further influenced by the interaction between vegetation cover, hunting strategy of the predator and anti-predatory strategy of the prey [20]. When more than one prey species is available, predators are able to shift prey selection depending on relative prey availability [21].

Here we investigate potential seasonality in the diet, prey preference and distribution of kills (i.e. feeding locations) of African lions (*Panthera leo*) in Hwange National Park, Zimbabwe, a semi-arid African savanna structured by artificial waterholes, where there is a strong seasonal variation in both surface-water availability and forage quality [22]. We assessed lion diet by combining carcass observations and faeces collection from GPS clusters, a method with recognized strengths [3], [4]. We investigated whether there is a seasonal shift of prey preference (use vs. availability) suggestive of different foraging strategies in different seasons. We expected lion kill locations to be influenced by the trend in prey aggregation at artificial waterholes, resulting in seasonal variation in kill distribution; closer to waterholes in dry periods and further away in wet periods.

## Materials and Methods

### Ethics Statement

All necessary permits were obtained for the described field study from the appropriate agency (Zimbabwe Parks and Wildlife Management Authority, 23(1) (c) (ii) 01/2005–2007).

### Study Area

The study was conducted between 2005 and 2007 in the northern sector of Hwange National Park (Hwange), north-western Zimbabwe, latitudes 18°30′ and 19°50′ S and longitudes 25°45′ and 27°30 E. The study area covers c. 7554 km^2^ of dystrophic woodland savanna. Altitude varies from 800 m to 1100 m and rainfall data were available from 1918 to 2007. Approximately 98% of the annual rainfall occurs from October to April and the long-term annual average (calculated for the period 1918–2007) is 632 mm (range: 324–1160 mm). Annual rainfall was 330 mm in 2004–2005, 683 mm in 2005–2006 and 703 mm in 2006–2007. There is no perennial water in Hwange, and rain fed pans hold water throughout the year only in high rainfall years. Water is artificially supplied to some waterholes during the dry season (∼40 in the study area). Three seasons are distinguished: the wet season (November-February), with long-term mean rainfall of 513.6±160.0 mm, wide spread surface-water availability and abundant, high quality browse and grazing; the early dry season (March-June), long-term mean rainfall of 111.1±72.8 mm with decreasing fodder quality and surface-water availability; and the late dry season (July–October), long-term mean rainfall of 25.0±26.6 mm with surface-water restricted to artificial waterholes and very few natural pans, while deciduous trees lose their foliage and both browse and grazing is of the lowest quality during the year.

### Lion Kill Data

Three male and eight female lions from different coalitions and prides, instrumented with GPS Simplex and TELUS radio-collars (approximate weights: female −900 g, male −950 g; Televilt Positioning AB, Lindesberg, Sweden; see [23] for details), were studied. Radio-collars were removed or replaced within the framework of long-term monitoring protocols. Positional data were recorded hourly during the night and retrieved at regular intervals. We systematically searched for potential lion kill sites by identifying clusters of x:y location coordinates including ≥4 hours of sequential locations within a defined proximity (150 m), and these were then investigated on foot (see also [4], [24]). Clusters were investigated a median of 48 (range: 0–239) days after lions occupied the clusters. We recognise that there may have been a small number of instances where lions scavenged other predators kills, however, we assume this to be negligible based on our kill site classification methodology. Kill sites were classified based on the forensic evidence of a kill. Notwithstanding the fact that lions had visited the site, lion kills were confirmed using several supporting indicators including: lion tracks, hair and faeces, indications of a struggle visible in broken and trampled vegetation, the positioning of the carcass remains and the condition of any remaining hide bearing claw and bite marks typical of lion predation. We initially identified 677 potential kill sites from the movement data of the 11 GPS collared lions (not all ≥4 hour clusters were initially identified, only the most recent at the time of the fieldwork), which were investigated on foot, and of which 245 were confirmed as lion kills (85 in the early dry season, 111 in the late dry season, and 49 in the wet season); other sites were often resting sites and therefore excluded, and any sites where scavenging was suspected were excluded as well. The kill sites were single kill events. Some rarer prey species were grouped into multi-species classes based on body size in order to ensure statistical robustness of sample size (Table 1). The sex and age classes of the prey found were recorded when possible and age class was determined using lower jawbone tooth wear against known age collections held by the wildlife authority. During the study period, adult male and female lions were sighted together in only 7% of the sightings (n = 1710). This is consistent with findings from Kruger National Park where GPS data from male and female lions suggest that males were only present with females at 10% of their kills [4]. Indeed, it has become apparent in Southern Africa that male lions hunt successfully and quite often in the absence of female lions [25], [26]. Hence male and female data were analysed separately.

**Table 1:** Lion prey species found at kills classified in 8 groups and their respective proportions relative to frequency of occurrence.

Species		Group	Number of Kills(♂/♀)	Proportion of total kills (♂/♀)	Seasonal proportion in kills		
Common name	Biological name				Early Dry (♂/♀)	Late Dry (♂/♀)	Wet (♂/♀)
Cape Buffalo	*Syncerus caffer*	***Buffalo***	45/55	0.56/0.33	0.53/0.27	0.59/0.36	0.54/0.36
Elephant	*Loxodonta africana*	Elephant	7/14	0.09/0.08	0.07/0	0.11/0.07	0.08/0.25
Giraffe	*Giraffa camelopardalis*	Giraffe	4/19	0.05/0.12	0.10/0.15	0.03/0.11	0/0.08
Greater Kudu	*Tragelaphus strepsiceros*	Kudu	4/26	0.05/0.16	0.07/0.22	0.05/0.14	0/0.11
Burchell’s Zebra	*Equus quagga*	Zebra	6/15	0.08/0.09	0.10/0.04	0.08/0.15	0/0.06
Roan Antelope	*Hippotragus equinus*	***Medium Bovidae***	9/22	0.11/0.13	0.10/0.22	0.05/0.08	0.31/0.11
Sable Antelope	*Hippotragus niger*						
Common Eland	*Taurotragus oryx*						
Wildebeest	*Connochaetes taurinus*						
Warthog	*Phacochoerus africanus*	Suidae	3/10	0.04/0.06	0/0.05	0.05/0.09	0.08/0
Bush pig	*Potamochoerus larvatus*						
Impala	*Aepyceros melampus*	Small Antelopes	2/4	0.03/0.02	0.03/0.05	0.03/0	0/0.03
Common Duiker	*Sylvicapra grimmia*						
Steenbok	*Raphicerus campestris*						
		Total sample size	80/165	80/165	30/55	37/74	13/36

### Lion Faecal Data

Samples were collected during kill site investigations, air dried and stored in paper bags for later analysis. Even though it is difficult to know from which individual a faecal sample comes from, and male and female lions are sometimes found together at a kill, samples were mainly collected at female kills and hence the faecal approach can be considered as more representative of female lion diet. Because several non-independent samples were collected at one site, we used one faecal sample only per kill site to avoid any pseuso-replication [27]. We used data from 74 faecal samples. Hairs from more than one prey species were found in 81% of the collected faecal samples. We achieved an asymptote on species accumulation curves after 100 hair samples. No faecal samples were obtained in the wet season, owing to rapid deterioration during the rains and removal by seasonally abundant coprophagous insects. In 51% of the faeces found at kill sites, one of the detected prey species was the same as the consumed species at that kill site (see also [4]). Samples were washed, sieved and sun dried to remove organic matter, and spread in a grid sampling tray. Following established methods, hair cross-sections and scale pattern imprints were prepared for microscopic analysis using a Watson Microsystem 70 microscope [28], [29]. For each hair the cross-section and a scale pattern was photographed and identified to species level using photographic reference libraries [30]–[33] as well as unpublished photographic reference libraries compiled from carcass, capture and museum specimen animals *in situ* in Zimbabwe. The prey items detected were categorised similarly to the prey detected from the kills. Ten hairs could not be identified (2% of all hairs collected).

### Prey Relative Contribution

First, seasonal importance of each species was assessed by (i) investigating, for lion males and females separately, the frequency of occurrence of prey species at kills, and (ii) comparing seasonal results from faecal data with no sex differentiation. Seasonal differences in the distribution of prey consumed were tested using chi-square tests. For kill data, we reported the relative contribution of each prey age class per season. We then converted the frequency of occurrence into biomass estimates using the average adult male and female mass (from [1], [34]). Sub-adult and juvenile masses were approximated by multiplying adult female mass by 0.7 and 0.3 respectively [1]. Where multi-species classes were defined, biomass was calculated as the average mass of the species included. Adult female mass for the different prey categories was 2275 kg for elephant (*Loxodonta africana*), 828 kg for giraffe, 513 kg for buffalo, 302 kg for zebra, 273 kg for the class “medium Bovidae” (see composition in Table 2), 157 kg for kudu (*Tragelaphus strepsiceros*), 56 kg for the class “Suidae” (see composition in Table 2), and 11 kg for “small antelopes” (see composition in Table 2). It was not possible to quantify biomass rigorously from faecal samples, as prey age and sex were not discernible from hair remains. We arbitrarily assigned the average adult female weight, with the exception of elephant as predominantly juveniles are preyed upon in dry periods [19], for each sample to allow a crude comparison with kill data.

**Table 2:** Lion prey species found in faecal samples classified in 10 groups and their respective proportions relative to frequency of occurrence.

Species		Group	Number and proportion of faecal samples with hair from the prey species			Proportion of the total number of prey found		
Common name	Biological name		Global	Early Dry	Late Dry	Global	Early Dry	Late Dry
Cape Buffalo	*Syncerus caffer*	Buffalo	29/0.39	14/0.39	15/0.39	0.18	0.18	0.18
Elephant	*Loxodonta africana*	Elephant	6/0.08	3/0.08	3/0.08	0.04	0.04	0.04
Giraffe	*Giraffa camelopardalis*	Giraffe	11/0.15	5/0.14	6/0.16	0.07	0.06	0.07
Greater Kudu	*Tragelaphus strepsiceros*	Kudu	39/0.53	19/0.53	20/0.53	0.24	0.25	0.24
Burchell’s Zebra	*Equus quagga*	Zebra	8/0.11	5/0.14	3/0.08	0.05	0.06	0.04
Roan Antelope	*Hippotragus equinus*	Medium Bovidae	29/0.39	13/0.36	16/0.42	0.18	0.17	0.19
Sable Antelope	*Hippotragus niger*							
Common Eland	*Taurotragus oryx*							
Wildebeest	*Connochaetes taurinus*							
Tsessebe*	Damaliscus lunatus*							
Waterbuck*	Kobus ellipsiprymnus*							
Reedbuck*	Redunca arundinum*							
Warthog	*Phacochoerus africanus*	Suidae	7/0.09	3/0.08	4/0.11	0.04	0.04	0.05
Bush pig	*Potamochoerus larvatus*							
Impala	*Aepyceros melampus*	Small Antelopes	27/0.36	13/0.36	14/0.37	0.17	0.17	0.17
Common Duiker	*Sylvicapra grimmia*							
Bushbuck*	Tragelaphus sylvaticus*							
Steenbok	*Raphicerus campestris*							
Climbing Mice*	Dendromus spp.*	Rodents*	4/0.05	2/0.06	2/0.05	0.03	0.03	0.02
Common Mice*	*Mus spp*.*							
		Total sample size	74	36	38	160	77	83

Species and groups marked with *were only recorded from faecal samples (not from kills).

### Prey Preference

We assessed seasonal prey preference using Jacobs’ selection index [35]:

D = r−p/r+p−2rp.

Where r is the proportion of the total number of kills or faecal samples of a particular species and p is the proportional availability of the prey species killed. Jacobs index ranges between −1 (highly avoided), 0 (used in proportion to availability) and 1 (highly selected). This index minimises the biases associated with small sample size (prey proportions below 10%), rare food items and non-linearity in proportional use over time [6]. Seasonal prey availability estimates for Hwange used in this analysis were taken from published road transect data for Sinamatella, Main Camp and Ngamo areas, which cover our study area [36]. Seasonal differences in the preference of prey were tested using Friedman rank sum tests.

### Role of Waterholes

Seasonal geographic information on waterholes was available and allowed us to calculate for each kill the distance to the nearest waterhole containing water (distance-to-water) using ArcView 3.2 nearest neighbour extensions (ESRI 2004). For each season, we also calculated the availability of each distance-to-water category in the study area. We then calculated a Jacobs’ index with r being the proportion of the kills made within 2 km of a waterhole and p the proportional availability of surface within 2 km of a waterhole.

## Results

### Prey Relative Contribution

Kill analysis revealed 14 different species being utilised by lions (Table 1). The most frequently occurring prey species were buffalo for both sexes: 56% for males and 33% for females. For males, buffalo was followed by the class “medium Bovidae” (11%) - class dominated by sable and wildebeest for kill data -, elephant (9%) and zebra (8%), together accounting for 84% of the prey detected (Table 1). For females, buffalo were followed by kudu (16%), the class “medium Bovidae” (13%), giraffe (12%), zebra (9%) and elephant (8%), together accounting for 91% of the prey detected (Table 1). Once biomass consumption was estimated, buffalo contributed the most to lion dietary intake (58% for males and 39% for females), followed by elephant (23% for males and 20% for females) and giraffe (7% for males and 18% for females), collectively accounting for 88% and 77% of the prey biomass consumed for males and females respectively (Table 3).

**Table 3:** Prey species proportional biomass contributing to lion diet, as detected from kills, faecal samples and observed hunts.

Species	Kill proportional biomass for males	Kill proportional biomass for females	Faecal sample proportional biomass	Observed hunt proportional biomass (Loveridge et al., 2006)
Buffalo	0.58	0.39	0.33	0.49
Elephant	0.23	0.20	0.09	0.16
Giraffe	0.07	0.18	0.20	0.09
Kudu	0.02	0.08	0.14	–
Zebra	0.04	0.05	0.05	0.05
Medium Bovidae	0.05	0.08	0.18	–
Suidae	0.004	0.01	0.01	–
Small Antelopes	0.001	0.001	0.01	–

**Figure 1:**
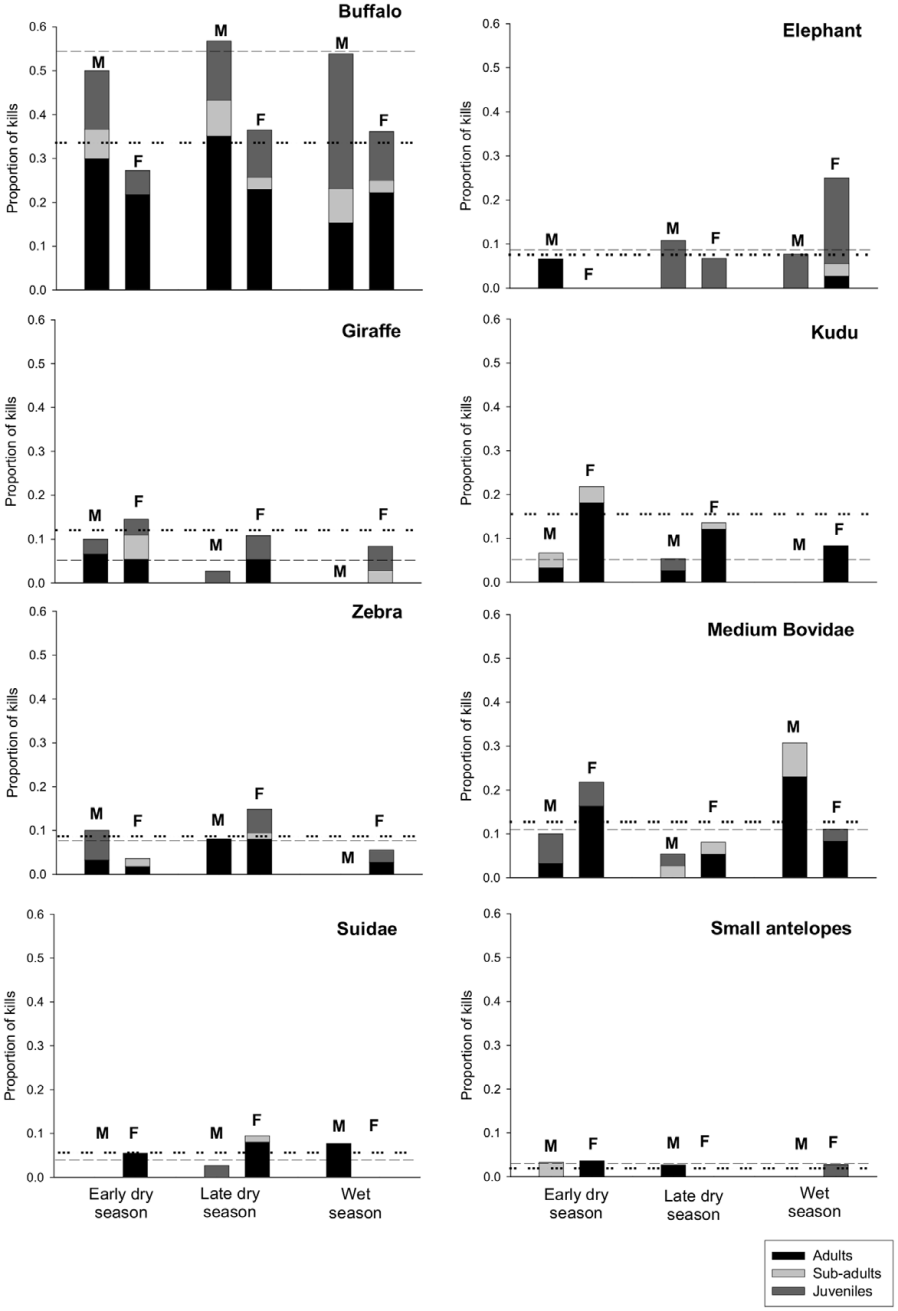
Seasonal variation in the relative contribution by different prey species to lion kills, for males (M) and females (F), distinguishing age class proportions. Dashed and dotted lines indicate mean proportions in the amalgamated kills for males and females respectively.

There was no seasonal difference in diet composition for male lions (χ^2^_early dry-late dry_ = 4.13, df = 7, p = 0.76; χ^2^_early dry-wet_ = 8.63, df = 7, p = 0.28; χ^2^_late dry-wet_ = 7.85, df = 7, p = 0.35). Female diet composition in the early dry season was significantly different from the other seasons (χ^2^_early dry-late dry_ = 19.06, df = 7, p = 0.008; χ^2^_early dry-wet_ = 20.34, df = 7, p = 0.005; χ^2^_late dry-wet_ = 14.07, df = 7, p = 0.05). This is due to the lower contribution of buffalo to female diet in the early dry season which is compensated by an increase in other species consumption (Table 1). Seasonal variation in relative contribution to lion kills by different prey species are shown in Fig. 1. Buffalo (the highest contribution in all seasons for both sexes) showed a constant contribution to male diet throughout the seasons, and a lower contribution to female diet in the early dry season. Proportions of each age class were relatively constant with an increase in the predation on juveniles by males in the wet season (Fig. 1). Zebra and Suidae showed a peak in contribution to female lion diet in the late dry season (Fig. 1). Overall, juveniles represented one third of the zebra carcasses found. Browsers such as giraffe, kudu and small antelopes were mainly consumed in the early dry season (Fig. 1). Juveniles also represented an important proportion of the giraffe killed by lions (Fig. 1). Medium Bovidae contributed least in the late dry season (Fig. 1). 62% of elephants were taken in 2005, which was a drought year (rainfall: 330 mm), concurring with increasing relative contribution of elephant to lion kills in the late dry season and peak contribution in the wet season (due to many kills in November before the first rains). Juveniles were taken almost exclusively (Fig. 1).

Faecal analysis revealed a higher number of species occurring in the lion diet (20 species), owing to the detection of more small prey items, unexpectedly including climbing mice (*Dendromus spp.*) and common mice (*Mus spp*.). The frequency of occurrence of species in the faecal dataset was highest for kudu (24%) followed by buffalo and medium Bovidae (each representing 18%; the medium Bovidae class is dominated by sable and eland for faecal data), small antelopes (17%), and giraffe (7%) collectively contributing 84% of the species found in faecal samples (Table 2). The remaining species contributed less than 5% per species. Once biomass consumption was estimated, buffalo contributed the most to lion dietary intake (33%), followed by giraffe (20%), medium Bovidae (18%), and kudu (14%) and collectively accounting for 85% of the prey biomass consumed (Table 3). Small antelopes contributed 1% to lion diet by relative biomass consumed. There was no seasonal difference in diet composition between the early and the late dry seasons (χ^2^ = 0.92, df = 8, p = 0.99).

### Prey Preference

**Figure 2:**
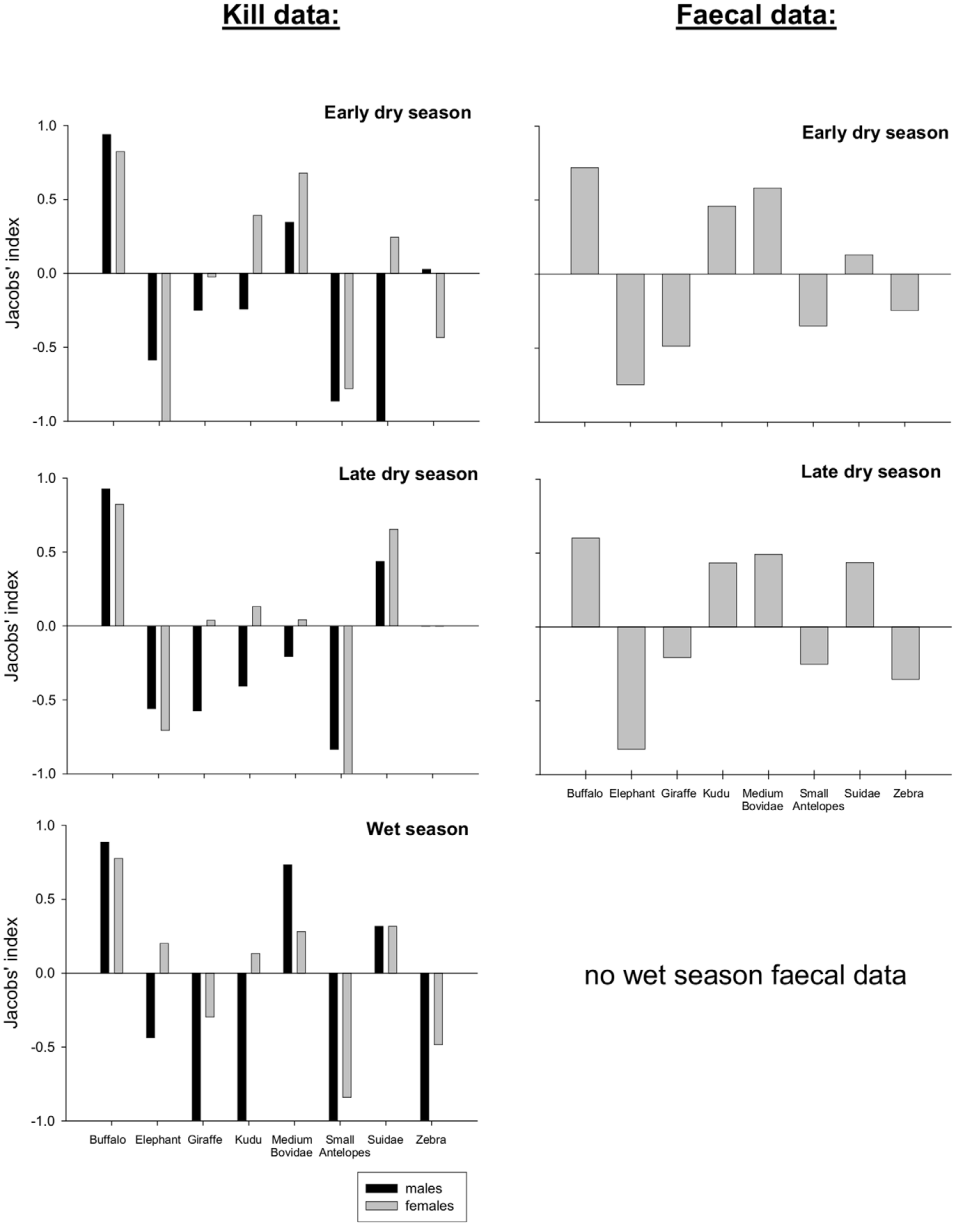
Jacobs’s index of seasonal prey preference estimated from confirmed lion kills and faecal samples. Values>0 indicate preference, values<0 suggest use but avoidance.

Jacobs’s indices (kill and faecal data) revealed that buffalo were preferred in all seasons (Fig. 2). Jacobs’s indices based on kills also revealed that female lions showed also a preference for kudu, medium Bovidae and Suidae in all seasons (Fig. 2). Male lions showed a preference for medium Bovidae in the early dry and wet seasons, and for Suidae in the late dry and wet seasons (Fig. 2). Overall, Jacobs’s indices showed avoidance of small antelopes and megaherbivores (elephant and giraffe) except for female lions which showed a slight preference for elephants in the wet season and consumed giraffe in proportion to their availability in the early and dry seasons (Fig. 2). Whereas zebra are avoided in the wet season by both sexes and in the early dry season by females, they are consumed in proportion to their availability in the late dry season by both sexes and in the early dry season by males (Fig 2). Jacobs’ indices based on faecal data are closer to those for females based on kill data, which is to be expected as most faecal samples were collected at female kill sites. Proportional prey selection did not differ significantly between seasons neither for males (Friedman χ^2^_early dry-late dry_ = 10.17, df = 7, p = 0.18; Friedman χ^2^_early dry-wet_ = 9.57, df = 7, p = 0.21; Friedman χ^2^_late dry-wet_ = 11.70, df = 7, p = 0.11), nor for females even though results approached significance level (Friedman χ^2^_early dry-late dry_ = 12.95, df = 7, p = 0.07; Friedman χ^2^_early dry-wet_ = 11.67, df = 7, p = 0.11; Friedman χ^2^_late dry-wet_ = 12.45, df = 7, p = 0.08).

### Role of Waterholes

**Figure 3:**
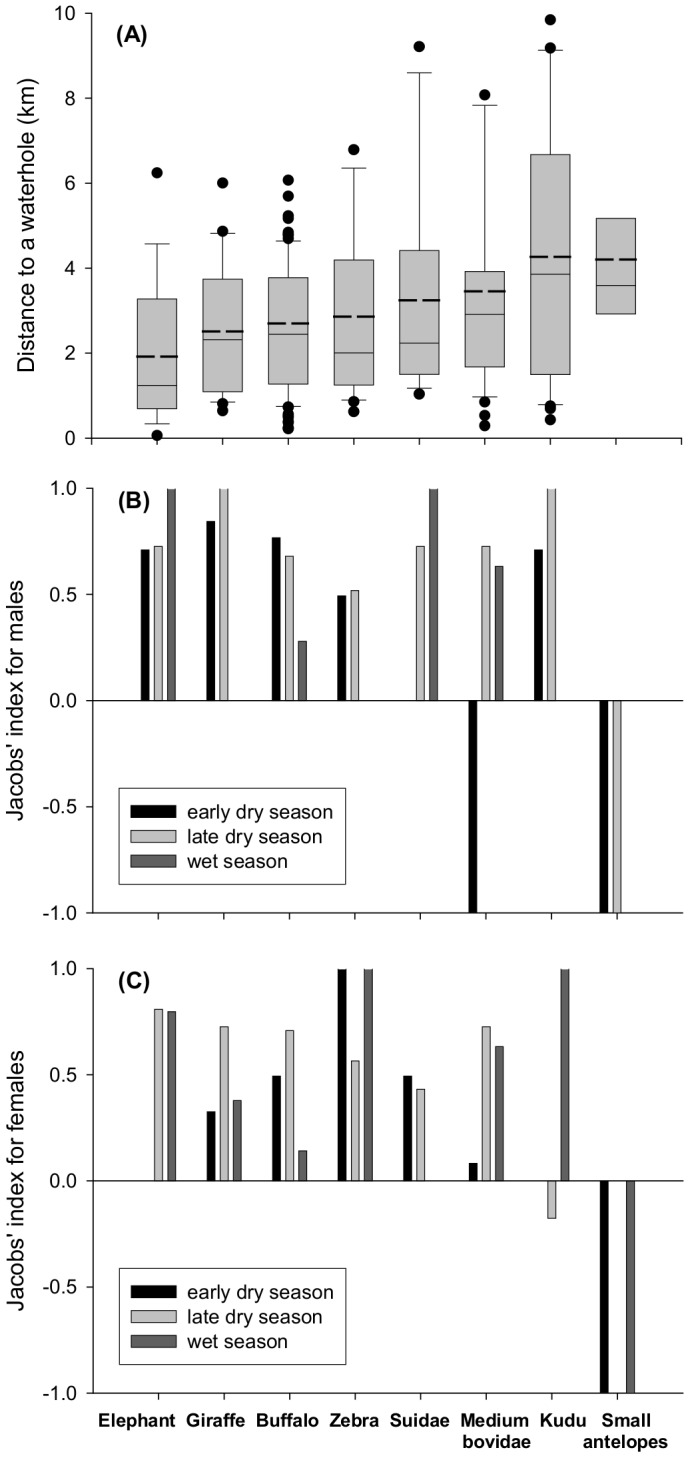
(A) Mean distance to a waterhole for kills for the different prey species. Boxes show medians, 25% and 75% quartiles. Bold dashed lines indicate means. Whiskers indicate the range between 10% and 90% percentiles. Dots represent data outside this range. **(B)** and **(C)** Jacobs’ index of seasonal preference to kill within 2 km of a waterhole for the different prey species for male and female lions respectively. Values >0 indicate preference, values <0 suggest use but avoidance.

Average distance-to-water revealed that lions typically kill between 1 and 4 km from a waterhole, with elephant and giraffe being the species killed most often the closest to a waterhole, and kudu and small antelopes killed the furthest away (Fig. 3A). Jacobs’ indices revealed that lions of both sexes kill their prey preferentially within 2 km from a waterhole for all prey species except small antelopes (Fig. 3B and C). For medium Bovidae in the early dry season, male lions killed the three prey recorded far from a waterhole (Fig. 3B).

## Discussion

Kill investigation to study carnivore diet is biased towards larger species that are easier to detect using GPS data since predators will stay longer at a the kill of a larger prey animal, and using carcasses alone clearly underestimates the number of feeding events on small species, sometimes up to 50% [3], [4]. In our case this is particularly true for the class “small antelopes”. In contrast, analysis of faeces tends to be biased against species with less hair, such as elephants, and very large bodied prey for which the body surface/volume ratio is smaller than for smaller species [37], and hence the likelihood of predators eating hairs is smaller. Here, we used a combination of the two approaches to provide the most complete description of lion diet in Hwange National Park, an approach that has already proved very useful for large carnivore diet in other systems [3]–[4], [38].

### Buffalo, the Primary Prey

Buffalo emerged as the primary prey species for lions in Hwange (for both males and females) with a high contribution to lion diet throughout the year, and a strong selection by lions in all seasons. The importance of buffalo was more pronounced for male lions, which corroborates findings from other studies [39]. Thus the importance of buffalo as prey is despite dry season variations when nutrition and water deprivation weaken susceptible individuals (young and old). Hence, buffalo are likely to have a crucial influence on the spatial and behavioural ecology of lions in the Hwange ecosystem in all seasons. In Hwange, previous findings have shown that lion home range size was influenced by buffalo density in the late dry season ([40]). Faecal data, mainly representative of female diet, suggest that kudu (revealed as the second main prey for females from kill data) may be an equally important prey species for female lions and that this species may be under-represented by kill data.

### Seasonality in Secondary Prey

Aggregation of herbivores at waterholes in the late dry season and wet season dispersion of herbivore herds, and particularly of buffalo herds, were expected to lead to pronounced seasonal foraging patterns for lions. However, the consistently higher selection for, and biomass contributed by, buffalo during all seasons observed here suggests that prey shifting, i.e. changing relative selection of prey species between seasons, is not characteristic of the Hwange lion population at least for the primary prey. No seasonal difference in male lion diet was detected, due to the prime importance of buffalo throughout the year. For females, some seasonal differences were detected due to the lower contribution of buffalo to female diet in the early dry season, compensated for by an increase in secondary prey consumption, mainly kudu and medium Bovidae. However, no significant shift in prey was revealed. Prey shift might be a response to larger variations in local prey abundance, perhaps over longer periods as shown for the Kruger National Park [21], or in ecosystems where more significant variation occurs in prey abundance over shorter time scales as shown for the Serengeti [41].

Some seasonal patterns were suggested for secondary prey, however. Overall, relative seasonal contribution revealed that grazers, such as Suidae and zebra, contribute more to lion diet in the late dry season when these species are heavily dependent on frequent access to surface-water. Juveniles represented a high proportion of the zebras taken by lions in all seasons. Given the propensity of kill investigations to be biased towards large prey, this is likely an underestimation and it should be considered that juvenile zebra are highly selected for by lions. Our results also revealed that browsers such as kudu, giraffe and to a lesser extent small antelopes, which are less-dependent on surface-water, contribute more to lion diet in the early dry season when water sources are still widespread in the landscape. Frequency of use (in both kills and faeces) and selection index of kudu in the early dry season highlight the importance of this species to lion female diet. This is consistent with previous findings suggesting that female lion home range size was constrained by the abundance of kudu in the early dry season [40].

### Opportunistic Foraging Behaviour

Our results showed that lions are opportunist hunters with prey ranging from mice to elephant. Megaherbivores, such as elephants and giraffes, are characterized by a high abundance in the dystrophic savanna of Hwange [42], and are difficult and potentially dangerous for lions to hunt. Nonetheless, kill investigations revealed that elephant is an important prey species, contributing 23% and 20% of biomass to male and female lion diet respectively. Furthermore, juvenile elephants were selected by female lions in the wet season. In our study, lions preyed mainly on elephant juveniles at the end of the dry season of a very dry year (October-November 2005), supporting previous findings that young elephants make up an unusually large proportion of lion prey in Hwange in the dry season during years of low rainfall [19]. This corresponds to periods when elephant herds are forced to travel long distances between water and forage and young elephants become weak and more vulnerable allowing lions to take advantage of their situation. Additionally, giraffe were consumed in proportion to their availability in the early and late dry seasons by female lions, and contributed 18% of biomass to female lion diet. Hence, lions appear to respond to the high abundance of megaherbivores in this ecosystem. Finally, faecal analyses revealed a significant proportional occurrence of small antelopes in lion diet (17%). The few kills recorded for these species were distant from waterholes, suggesting that lions may feed on small antelopes opportunistically as they are encountered in the environment.

### Importance of Waterholes in Lion Foraging Behaviour

Lion kills were located in a preferentially selected “zone” around artificial waterholes, suggesting that these scarce resources form passive traps for ungulate prey. Lions are stalk-and-ambush hunters that use vegetative cover for concealment during hunting [43] and are known to ambush prey in habitats surrounding high-prey abundance areas [44]. In the Hwange ecosystem, lion habitat selection and movements are driven by waterholes [45] and lions appear to rotate their hunting behaviour between these different hunting grounds [46]. Water sources are also considered crucial in lion habitat selection in the Serengeti [47] and thought to act as passive traps for ungulates in the Kruger ecosystem [5], [48]. Contrary to our predictions, areas close to waterholes were highly selected for kills regardless of seasonal conditions. In Hwange, the vegetation is primarily woodland and bushland and open grassland areas are scarce but often associated with waterhole areas [49]. Consequently, waterhole areas are attractive for all herbivores in the dry season and attractive for grazers in the wet season, which corresponds to the period when most grazers have their young. Artificial waterholes act as powerful hubs of predation activity throughout the year. Understanding how predators make use of their environment and how actions such as augmenting water supply can alter their behavioural ecology, has implications for the long term sustainability of predator-prey systems. This is reflected by the declines in certain rare herbivores when artificial water points allowed more common herbivores and resident predators access to areas previously too dry to support them [48], [50].
